# Fischer Rats Consume 20% Ethanol in a Long-Term Intermittent-Access Two-Bottle-Choice Paradigm

**DOI:** 10.1371/journal.pone.0079824

**Published:** 2013-11-14

**Authors:** Douglas J. Mill, Jade J. Bito-Onon, Jeffrey A. Simms, Rui Li, Selena E. Bartlett

**Affiliations:** 1 Translational Research Institute and Institute for Health and Biomedical Innovation, Queensland University of Technology, Brisbane, Queensland, Australia; 2 Ernest Gallo Clinic and Research Center at the University of California San Francisco, Emeryville, California, United States of America; University of Insubria, Italy

## Abstract

The 20% ethanol intermittent-access (IAE) two-bottle-choice drinking procedure has been shown to produce high voluntary ethanol consumption in a number of rat strains. For this study, we applied this procedure to male Fischer (F344) rats, a strain previously reported to exhibit low levels of ethanol consumption. We also subjected these animals to a two-week ethanol-deprivation-period to see if they would exhibit an alcohol deprivation effect (ADE) signified by a transient increase in alcohol consumption following deprivation. Our data show a separation between high and low consuming animals within this strain, with high-consumers exhibiting an escalation in consumption. In contrast, Fischer rats did not show a significant separation between high and low consumers or any significant escalation in consumption, using the 20% ethanol continuous-access two-bottle-choice drinking protocol. Following the two-week deprivation period, animals in the high (but not the low) IAE group exhibited the transient increase in ethanol consumption and preference typically associated with an ADE. Together, the data suggest that the intermittent access protocol is a useful protocol for increasing ethanol consumption.

## Introduction

Preclinical models play an integral role in the development of therapeutics for the treatment of alcohol use disorders, bridging the gap between laboratory and clinical research and helping to elucidate the mechanisms underlying both the development of alcoholism and the utility of the medications themselves. The intermittent-access 20% ethanol (IAE) two-bottle-choice drinking procedure has proven to be a reliable and efficacious model for creating high levels of voluntary oral ethanol consumption in Long-Evans, Wistar, and Sprague-Dawley rats [1]-[4]. In the present study, we determined whether the IAE procedure would be effective in Fischer (F344) rats, a strain shown to demonstrate a low preference for alcohol and thus rarely utilized in studies examining ethanol-reinforced behaviors [5]–[7]. Additionally, we subjected these animals to a two-week alcohol deprivation period in order to determine if F344 rats will demonstrate an alcohol deprivation effect (ADE), which is characterized by a transient increase in alcohol consumption and/or preference following deprivation. The ADE has been studied extensively in other rat strains and is postulated to be a model of craving and compulsive ethanol-seeking [8]–[11].

## Materials and Methods

Adult, male, ethanol-naïve, Fischer (F344) rats weighing 150–175 g upon arrival (Charles River, Wilmington, MA USA), were individually housed in ventilated Plexiglas cages (Thoren Caging Systems Inc., Hazelton, PA, USA) in a climate-controlled room on a 12-h reverse light/dark cycle (lights off at 10 a.m.). Rats were given at least one week to acclimate to individual housing conditions and handling procedures. Food and water were available *ad libitum* in the home cage throughout the entire experiment. All procedures were pre-approved by the Ernest Gallo Clinic and Research Center Institutional Animal Care and Use Committee and were in accordance with the NIH *Guide for the Care and Use of Laboratory Animals*. Ethanol solutions were prepared in filtered water using 95% (v/v) ethanol (Gold Shield Chemical Co., Hayward, CA, USA). All fluids were presented in 100-ml graduated glass cylinders with stainless-steel drinking spouts inserted through two grommets in front of the cage 10 min after the lights went off in the reversed light/dark cycle room. Bottles were weighed 24 hours after the fluids were presented, and measurements were taken to the nearest tenth of a gram. Spillage from the bottles was negligible and amounted to less than 0.5 grams per bottle per 24 hour access period. The weight of each rat was measured daily Monday through Friday to monitor health and calculate the grams of ethanol intake per kilogram of body weight. Ethanol preference was calculated by dividing the volume of 20% ethanol consumed by the volume of total fluid (water + ethanol) consumed, and is expressed as a percentage.

To determine whether the IAE protocol would produce high voluntary ethanol intake in F344 rats, two groups (n = 24 total) were trained to consume 20% ethanol. The rats were given access to one bottle of 20% ethanol and one bottle of water for three 24-hour-sessions per week (Mondays, Wednesdays and Fridays) as adapted from [3] and previously described [1]. The rats had unlimited access to two bottles of water between the ethanol-access periods. The placement of the ethanol bottle was alternated each ethanol drinking session to control for side preferences. We maintained the rats on the IAE procedure for a total of 52 ethanol-access sessions (44 sessions pre-deprivation and 8 sessions post-deprivation). No sucrose fading technique was employed in this initiation phase.

Following 15 weeks of the IAE procedure, animals underwent a two-week ethanol deprivation period (equal to six ethanol sessions). Throughout the deprivation period, food and water were available *ad libitum* at all times in the home cage with a second water bottle placed into the vacant grommet. The weight of each rat was measured weekly to monitor health. At the end of the two week deprivation period, all animals were re-exposed to the IAE procedure and measured for both ethanol preference and ethanol and water intake.

For comparison of baseline consumption, a separate group (n = 12) of F344 rats was maintained on a continuous-access 20% ethanol protocol for nine weeks in order to compare their voluntary ethanol consumption to that of the animals maintained on the IAE protocol. The rats were given access to one bottle of 20% ethanol and one bottle of water 24 hours a day, seven days a week for the duration of the experiment. No sucrose was used to initiate drinking. Ethanol and water bottles were weighed 4-5 times per week (total of 40 sessions with the bottles weighed) to calculate ethanol intake and preference. Animal weights were also recorded on these days. The placement of the ethanol bottle was alternated each day to control for side preferences.

Statistical analysis was performed using SigmaStat version 3.5 (Systat Software, San Jose, CA) and GraphPad Prism 5 (GraphPad Software, Inc., La Jolla, CA). The behavioral data were analyzed using one or two-way ANOVA where appropriate, followed by Newman-Keuls post hoc analysis when a significant overall main effect was found (*p*<0.05). In order to study the differences in ethanol consumption and escalation between high and low ethanol-consuming animals within the IAE and continuous access groups, a median split was performed to separate the animals into two equal groups for each of the training schedules based on the average ethanol consumption (g/kg) for the last three ethanol exposures (i.e., n = 12 per group for IAE and n = 6 per group for continuous access).

## Results

**Figure 1:**
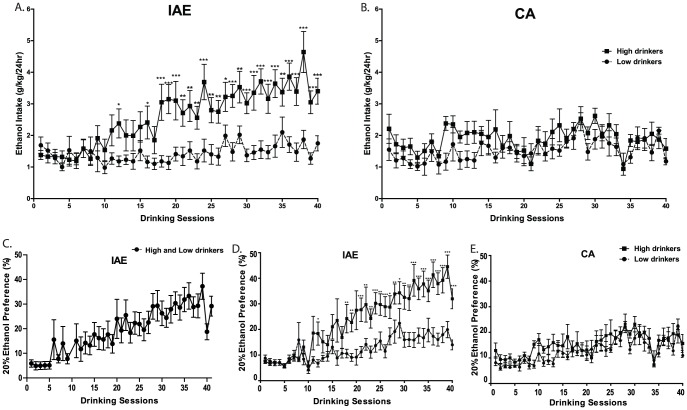
Fischer F344 rats have increased ethanol consumption and preference using the intermittent access to 20% ethanol procedure. Escalation of 20% ethanol (**A**) consumption and (**C-D**) preference in F344 rats using the intermittent access to 20% ethanol (IAE) procedure. Animals were divided into groups of high-drinkers (n = 12) and low drinkers (n = 12) with the high group exhibiting significantly higher ethanol consumption. (C) Ethanol preference for the combined high and low drinkers trained on the IAE procedure. F344 rats on a 20% ethanol continuous-access (CA) schedule showed no significant difference between high and low groups for (**B**) consumption or (**E**) preference and no significant escalation for either measure overall. The values are expressed as mean ethanol intake (g ( kg ( 24 h) or preference (ratio of ethanol over total fluid intake) ± SEM at each drinking session. *p< 0.05, **p<0.01, and ***p<0.001 comparing the two groups (two-way repeated measures ANOVA followed by Newman–Keuls post hoc test).

**Figure 2:**
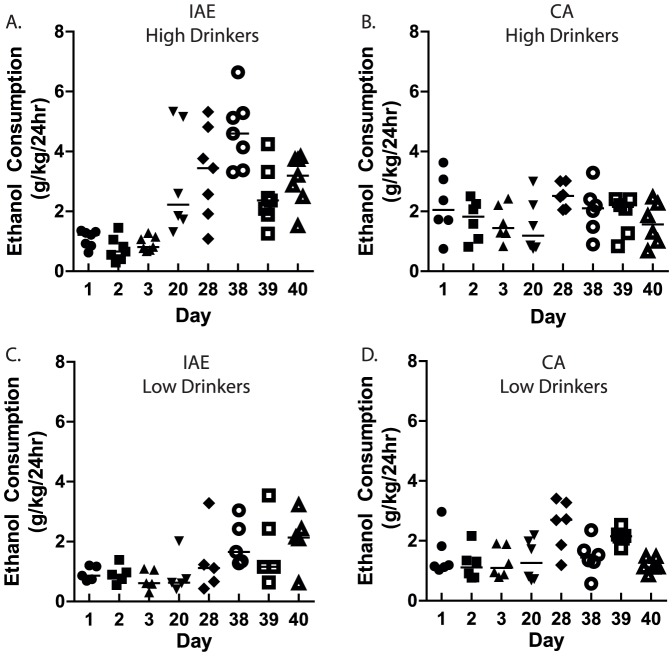
Individual ethanol intake values in F344 rats using the intermittent access (IAE) and the continuous access (CA) procedures. Animals were divided into groups of high-drinkers (**A-B**) and low drinkers (**C-D**) and ethanol intake values plotted for the IAE procedure (**A, C**) and the CA access procedure (**B, D**) for sessions on Days 1, 2, 3, 20, 28, 38, 39, 40. The values are expressed as mean ethanol intake (g ( kg ( 24 h) at each drinking session.

Analysis of the ethanol consumption for the two groups of F344 rats (n = 24) trained on the IAE schedule failed to reveal the typical escalation in drinking exhibited by other rat strains in our previous studies. A one-way repeated measures ANOVA analysis of the ethanol consumption for the entire population revealed a significant overall effect of day [*F*(39, 956)  = 6.77, *p*<0.001]. Post hoc analysis revealed that while drinking was elevated for some of the drinking sessions, there was no period of sustained escalation. We, therefore, performed a median split on the data from the IAE groups based on the average ethanol consumption for the last three ethanol exposures prior to deprivation in order to study the differences in ethanol consumption and escalation between high and low ethanol-consuming animals. Beginning in the eighth week of ethanol exposure, a significant escalation in ethanol drinking began in the high, but not the low, consuming rats. A two-way repeated measures ANOVA analysis comparing the daily ethanol consumption (g/kg/24hr) of the two groups revealed an overall main effect of group [*F*(1, 956)  = 14.50, *p<* 0.001], an overall main effect of day [*F*(39, 956)  = 7.84, *p*<0.001], and an overall significant interaction (group x day) [*F*(39, 956)  = 4.55, *p*<0.001]. Post-hoc analysis (Newman-Keuls) revealed significant differences in consumption between the groups and starting with session 18 the high group exhibited significantly higher ethanol consumption for the remainder of the experiment (Fig. 1A). Within the high group, daily ethanol consumption was significantly escalated beginning at session 24 when compared to the initial intake level (session 1). The low group did not exhibit significant escalation in daily ethanol consumption (Fig. 1B). We measured the ethanol preference for the F344 rats trained on the IAE schedule (Fig. 1C). Two-way repeated measures ANOVA comparing the ethanol preference for the high ethanol and low ethanol drinking groups trained on the IAE schedule revealed an overall main effect of group [*F*(1, 929)  = 12.55, *p<* 0.01, Fig. 1D], an overall main effect of day [*F*(39, 929)  = 16.89, *p*<0.001], and an overall significant interaction (group x day) [*F*(39, 929)  = 4.43, *p*<0.001]. Post-hoc analysis (Newman-Keuls) revealed significant differences in preference between the groups and starting with session 18 the high group exhibited significantly higher ethanol preference for F344 rats trained on the IAE schedule (Fig. 1D). Within the high group, daily ethanol preference was significantly escalated beginning on session 21 when compared to the initial ethanol preference level (session 1). The low group did not exhibit significant escalation in daily ethanol preference (Fig. 1D). There was no significant difference in body weight between the groups over the course of the study (data not shown), and there were no visible adverse health effects of ethanol consumption in the high-consuming group. A separate group (n = 12) of F344 rats was maintained on a continuous-access to 20% ethanol schedule for nine weeks in order to compare their voluntary ethanol consumption to that of the F344 rats on the IAE schedule. After performing a median split on the data from the continuous access animals based on the same criteria as described above for the IAE group, a repeated-measures two-way ANOVA revealed no significant difference between the high and low groups for ethanol consumption (F(1,477)  = 3.96, n.s., Fig. 1B) or preference (F(1,476)  = 3.44, n.s., Fig. 1E). Repeated-measures two-way ANOVA also showed that both the high and the low ethanol consuming groups using the continuous access to ethanol procedure did not have any significant escalation in consumption or preference over the course of the experiment (Fig. 1B and 1E). To show the inter-animal variability between the high and low ethanol consumption using the intermittent and continuous access procedures, the individual ethanol intake values were plotted and are expressed as mean ethanol intake (g / kg ( 24 h) at each drinking session on days 1, 2, 3, 20, 28, 38, 39, 40 for the high (Fig. 2A&B) and the low drinkers (Fig. 2 C&D).

**Figure 3:**
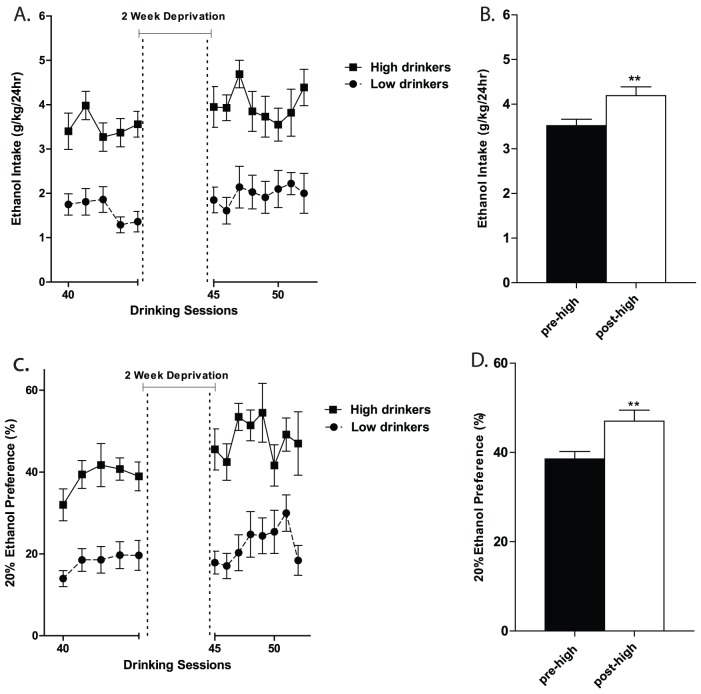
Ethanol consumption and preference following a two week deprivation period. (**A**) 20% ethanol consumption in F344 rats trained using the IAE procedure before and after a two-week deprivation period. (**B**) Animals in the high group exhibited a significant escalation in ethanol intake following a two week deprivation period. (**C**) Ethanol preference before and after a two-week deprivation period. (**D**) Animals in the high group exhibited a significant increase in ethanol preference following deprivation. The values are expressed as mean ethanol intake (g/kg/24h) or preference (ratio of ethanol over total fluid intake) ± SEM. To calculate the ADE effect the five drinking sessions preceding and three drinking sessions following deprivation were analyzed by one-way ANOVA, **, p<0.01 comparing pre- and post-deprivation periods.

Following the two-week deprivation period, animals in the high (but not the low) IAE group exhibited the transient increase in ethanol consumption and preference typically associated with an ADE. To study the ADE, we performed statistical analyses on the five drinking sessions preceding and three drinking sessions following the two-week deprivation period. Two-way repeated measures ANOVA comparing the daily ethanol consumption (g/kg/24hr) and preference (%) from the two groups revealed overall main effects of group [F(1,308)  = 27.20, p<0.001 for consumption and F(1,308) = 27.29, p<0.001 for preference: Fig. 3A and 3C, respectively], and day [F(12,308)  = 3.52, p<0.001 for consumption and F(12,308)  = 5.67, p<0.001 for preference], but no significant overall interactions. To further explore the ADE, we performed a one-way ANOVA comparing the five sessions immediately preceding and three sessions immediately following the deprivation period for each of the groups (low and high). Within the high group, daily ethanol consumption was significantly higher following deprivation [F(1,94)  = 7.92, p<0.01: Fig 3B]. Daily ethanol preference within the high group was also significantly higher following deprivation [F (1,93)  = 8.70, p<0.01: Fig 3D]. Within the low group, neither daily ethanol consumption nor preference was significantly different following deprivation (F (1,95)  = 1.46, n.s. for g/kg consumption and F(1,95)  = 0.02, n.s. for preference).

## Discussion

Our results demonstrate that a subpopulation of F344 rats will significantly escalate 20% ethanol consumption using the IAE procedure. The distinct separation between the escalation curves of the high and low consuming animals is of particular interest as it has not been observed in other rat strains exposed to the IAE procedure, where a majority of the animals exhibit high ethanol consumption [1], [2]. There are many ethanol self-administration studies have relied upon comparisons between genetically selected high-preferring and non-preferring lines (P vs. NP, sP vs. sNP, AA vs. ANA, or HAD vs. LAD rats) in order to examine phenotypic differences between high and low ethanol-preferring animals [12]–[15]. We have previously shown that intermittent access to ethanol induces an increase in ethanol consumption in Wistar, Long-Evans and P-rats [1], [2]. However, in Fischer rats only a sub-group showed an escalation in ethanol intake following the intermittent access protocol. As the intermittent access protocol, unlike the continuous access procedures, gives the animals days off from ethanol, this is akin to a withdrawal period. We speculate that the reason there are individual differences in the level of ethanol intake following the intermittent access procedure may be related to baseline differences in the animal’s anxiety and/or stress levels, however this remains to be investigated.

It is important to note that the escalation of ethanol intake in the high group was much more gradual than typically observed for Long-Evans and Wistar rats. Our high-consuming animals were not significantly escalated until the 24th ethanol exposure, whereas Long-Evans and Wistar rats have been shown to significantly escalate their ethanol consumption by sessions 5–10 [1]. Previous ethanol self-administration studies utilizing F344 rats showed very low ethanol preference in this strain [5]–[7]. While F344 rats still consume significantly less ethanol than other strains within this model, our results indicate that splitting F344 rats into groups of high and low drinkers reveals a significant escalation of ethanol consumption within the high-preferring animals. In fact, the consumption and preference levels among the three highest-consuming animals approach those found on average for other strains [1], [2]. In contrast to F344 animals trained on the IAE schedule, F344 rats trained using a 20% ethanol two-bottle-choice continuous-access schedule did not exhibit a significant difference between high and low consuming groups and showed no significant escalation of consumption. This finding indicates that the IAE procedure may be effective technique to increase ethanol intakein some low-consuming rat strains.

In addition, following a two-week deprivation period from ethanol, the high-drinking F344 rats on the IAE schedule, the animals’ further escalated their ethanol intake. Previous studies have examined the ADE as a model of relapse and/or craving, and, furthermore, the manifestation of increased alcohol consumption following a period of abstinence has been documented in humans as well as rodents [10], [11]. Human alcoholics will often demonstrate repeated cycles of abstinence and relapse, with each relapse period driving their alcohol consumption higher and decreasing their responsiveness to treatment [10]. Furthermore, the ADE is not thought to be a means of compensating for withdrawal as it persists long after physical withdrawal symptoms are gone and occurs in non-dependent animals and humans. The present data also indicate that these changes are associated with the amount of ethanol consumed during the initial 44 sessions was not observed in the low-drinking IAE animals. Future studies utilizing more deprivation periods and/or deprivation periods of different lengths within this model on F344 rats could help to uncover differences between rat strains. A study of 20% ethanol operant self-administration could also be beneficial in allowing one to compare motivated responding for 20% ethanol to that of other strains. We also did not examine whether the animals that had continuous access to ethanol would increase drinking following a two week deprivation period, as the level of ethanol consumption was very low. Therefore we cannot rule out the drug access conditions themselves may contribute to the escalation in drinking rather than the ethanol intake. This study shows that a subpopulation of F344 rats can be trained to self-administer 20% ethanol using the IAE model and that this subpopulation demonstrates an ADE. These findings indicate that F344 rats are interesting candidates for future studies examining within-strain differences in alcohol consumption.
